# Colorimetric and Electrochemical Screening for Early Detection of Diabetes Mellitus and Diabetic Retinopathy—Application of Sensor Arrays and Machine Learning

**DOI:** 10.3390/s22030718

**Published:** 2022-01-18

**Authors:** Georgina Faura, Gerard Boix-Lemonche, Anne Kristin Holmeide, Rasa Verkauskiene, Vallo Volke, Jelizaveta Sokolovska, Goran Petrovski

**Affiliations:** 1 Center for Eye Research, Department of Ophthalmology, Institute of Clinical Medicine, Faculty of Medicine, University of Oslo, Kirkeveien 166, 0450 Oslo, Norway; g.f.munoz@medisin.uio.no (G.F.); g.b.lemonche@medisin.uio.no (G.B.-L.); 2 Department of Medical Biochemistry, Institute of Clinical Medicine, University of Oslo, 0424 Oslo, Norway; 3 Sharelab, Biozep AS, Oslo Science Park, Gaustadalleen 21, 0349 Oslo, Norway; ak.holmeide@biozep.com; 4 Institute of Endocrinology, Medical Academy, Lithuanian University of Health Sciences, LT-50009 Kaunas, Lithuania; rasa.verkauskiene@gmail.com; 5 Department of Physiology, Institute of Biomedicine and Translational Medicine, University of Tartu, 19 Ravila Street, 50411 Tartu, Estonia; vallo.Volke@kliinikum.ee; 6 Institute of Biomedical and Transplant Medicine, Department of Medical Sciences, Tartu University Hospital, L. Puusepa Street, 51014 Tartu, Estonia; 7 Faculty of Medicine, University of Latvia, Jelgavas Street 3, LV 1004 Riga, Latvia; jelizaveta.sokolovska@lu.lv; 8 Department of Ophthalmology, Oslo University Hospital, 0450 Oslo, Norway

**Keywords:** diabetes mellitus, diabetic retinopathy, screening, early detection and diagnosis, point-of-care, glucose sensing, sensor arrays, machine learning

## Abstract

In this review, a selection of works on the sensing of biomarkers related to diabetes mellitus (DM) and diabetic retinopathy (DR) are presented, with the scope of helping and encouraging researchers to design sensor-array machine-learning (ML)-supported devices for robust, fast, and cost-effective early detection of these devastating diseases. First, we highlight the social relevance of developing systematic screening programs for such diseases and how sensor-arrays and ML approaches could ease their early diagnosis. Then, we present diverse works related to the colorimetric and electrochemical sensing of biomarkers related to DM and DR with non-invasive sampling (e.g., urine, saliva, breath, tears, and sweat samples), with a special mention to some already-existing sensor arrays and ML approaches. We finally highlight the great potential of the latter approaches for the fast and reliable early diagnosis of DM and DR.

## 1. Introduction

Diabetes mellitus (DM) is a group of metabolic diseases involving severe insulin deficiency with usually acute onset of hyperglycemia due to autoimmune destruction of pancreatic beta cells, or gradual onset of hyperglycemia due to insulin resistance [1]. Diabetic retinopathy (DR) is a complication of DM, and the main cause of blindness in working-age adults, which can be retarded, palliated, or even avoided if detected early. Unfortunately, most patients who develop this condition are asymptomatic until late stages, when treatment is less effective or DR is irreversible. Classically, screening procedures for DR are usually based on imaging techniques of the fundus of the eye. The analysis of such images has been optimized during the last years by machine learning (ML) methods, capable of detecting even microaneurysms—the earliest visible sign of retinal damage [2,3]. The relatively recent application of user-friendly and reasonably affordable smartphones for the implementation of computer-assisted approaches has permitted the exportation of such screening imaging-based methods to developing countries, where lack of funds and personnel have often been the limiting factors to their healthcare systems [4,5,6,7]. Many recent reviews have already extensively explored the possibilities of computer-assisted methods for screening DM and DR [4,7,8,9,10,11,12,13,14,15], which still present few actual applications in the form of commercially available products [4]. Less (but commendable) attention is paid to alternative and early DR detection techniques, such as electrochemical [16] or colorimetric [17,18,19]. These methods usually rely upon the detection of a biomarker (or multiple biomarkers simultaneously) which might indicate the presence of the target disease (potentially earlier than the image-based methods, which usually rely upon observation of already-damaged tissues) [20]. The higher reliability of these multi-targeted sensing approaches can even be improved by the use of technologies with multiple and diversely integrated sensors, for which the great amount of data can be efficiently filtered and processed by ML. In this review, we present a series of colorimetric (with special emphasis on naked-eye approaches) and electrochemical techniques for the non-invasive detection and/or quantification of DM biomarkers, including sensor arrays powered or not by ML models [21]. The scope is to encourage the reader towards development of ML-powered sensor arrays for early diagnosis of DM and DR.

## 2. Previous Considerations

### 2.1. The Relevance of Early Detection of Type 2 DM

It has been observed that a subset of patients with type 2 DM (T2D) have signs of DR at the time of diagnosis; moreover, glucose intolerance or pre-diabetes is also associated with diabetic eye disease [22,23]. Therefore, improvement and accessibility of the screening for DM is directly related to the screening and identification of DR. Although DR prevalence seems to be higher and develops faster in type 1 DM (T1D) patients, it is more difficult to prevent DR in T2D patients, as its progression strongly depends on the duration of DM. T2D often remains undiagnosed for a longer time (even several years) compared to T1D [24]. Moreover, T2D is much more common than T1D (i.e., approximately 90% of the total DM cases) [25]. Consequently, a special emphasis has to be placed on the screening of T2D, provided the higher impact of the early detection of this type of DM and, therefore, prevention and mitigation of DR.

### 2.2. Non-Invasive Sampling

Even if blood testing is one of the most used methods in diagnosis of DM [26,27,28,29], great efforts have been made during the last century in the study of non-invasive testing alternatives [30]. Blood tests present risk of infection and require skilled personnel during both sampling and analysis. Moreover, multiple extractions are painful and generate fear and anxiety to some patients, which can lead to avoidance behaviors, with the subsequent public health and social consequences [31]. Thus, the use of other physiological fluids, which do not imply invasive techniques per se, are indeed desirable. Hence, the fluids considered in this review are urine, saliva, tears, sweat, and breath, which contain biomarkers relevant for the diagnosis of DM and DR (some examples in Table 1) and have already been studied in terms of sensing [30,32,33]; these have turned out to be promising alternatives for the development of less painful and more economic methods as compared to blood testing.

### 2.3. The Great Potential of Sensor Arrays

All physiological fluids are complex matrices with many potential interfering factors, which are concomitant part of the heterogeneous nature of DM (depending on, for instance, the patient) [25]. Consequently, the study of a single biomarker in a fluid can easily lead to many technical issues, with the results obtained being false positive or negative. In order to overcome this problem, we propose the design of sensor arrays, based on diverse principles (e.g., chemical, enzymatic, pH or immunoassay-based), as well as the inclusion of diverse biomarkers. This approach can provide a much more meaningful set of qualitative/quantitative information, which would significantly improve the accuracy of the screening, leading to a more robust and reliable interpretation of the results. The multi-marker approach is not new in the diagnostics area [25,34], but simpler, faster, and cheaper methods are needed for effective screening of DM and the consequent minimization of the related retinal damage in these patients. The designed sensor array should present the so-called ASSURED characteristics (affordable, sensitive, specific, user- friendly, rapid and robust, equipment free, and deliverable to end-users) as described by the World Health Organization, and they should permit their application as point-of-care (POC) screening devices [35].

### 2.4. The Important Role of ML

Sensor arrays can generate a great amount of data that, when correctly interpreted, provide filtered and relevant information. Such a great amount of data cannot always be understood intuitively by applying simple mathematical models—e.g., linear or polynomial regressions [36]. However, usually a hidden pattern explains the observed data, even if we ignore the exact analytes and/or mechanisms that generate the output. By using ML approaches, it is possible to bypass the need for deep understanding of the hidden rules underlying the studied system, but still getting relevant information for the prediction of trends, groups, and characteristics [37,38]. ML models have proved to be robust for the diagnosis of several diseases [39,40,41,42,43], including ophthalmology-related ones [4,14]. In the literature, we can find numerous examples of ML-sensor-array technologies for diagnostics, from techniques to detect lung cancer [44] to multi-sensors capable of diagnosing respiratory diseases and breast cancer from breath air [45,46], which proves the great potential of these approaches for pre-clinical diagnosis, screening purposes, and to assist practitioners in making fast decisions [46]. The miniaturization of such devices and the optimization of the related production process could lead in the near future to the fabrication of POC sensor-array systems, with coupled software trained to diagnose a disease or condition (or even more than one) by simple non-invasive analysis of the breath, urine, saliva, tears, and/or sweat of the patient.

**Table 1:** Biomarkers related to DM and DR found in urine, saliva, sweat and tears, along with some commercial kits exploiting them.

Biomarkers	DM	DR	Commercial Kits	References
**URINE**				
**Glucose**	X	X	URINSTIX 10 (Test Helsen SA) Glucose assay kit (Merck)	Corrie et al. [47] Makaram et al. [48]
**1,5-anhydro-d-glucitol (1,5-AG)**	X	X	-	Yamanouchi et al. [49]
**Microalbuminuria**	X		Microalbustix (Bayer)	Mogensen et al. [50]
**β-hydroxybutyric acid (βHBA)**	X		β-Hydroxybutyrate (Ketone Body) Colorimetric Assay Kits (Cayman Chemical)	Sacks et al. [51]
**Acetoacetate (AcAc)**	X		URINSTIX 10 (Test Helsen SA)	Sacks et al. [51]
**SALIVA**				
**Glucose**	X	X	Glucose assay kit (Merck)	Gupta et al. [52] Baliga et al. [53] Jenkins et al. [20]
**Lactate**	X		Lactate assay kit (Merck)	Deng et al. [54] Calabria et al. [55] Jenkins et al. [20]
**1,5-AG**	X		Glycomark^®^ assay kit (FDA approved) *	Halama et al. [56]
**SWEAT**				
**Glucose**	X	X	Glucose assay kit (Merck)	Lee et al. [57] Heikenfeld et al. [58]
**TEARS**				
**Glucose**	X	X	Glucose assay kit (Merck)	Badugu et al. [59,60] Corrie et al. [47]
**Lipocalin 1 (LCN-1)**		X	-	Csősz et al. [61] Kim et al. [62]
**Lactotransferrin,**		X	-	Csősz et al. [61]
**Lacritin**		X	-	Csősz et al. [61]
**Lipophilin A**		X	-	Csősz et al. [61]
**Lysozyme C**		X	-	Csősz et al. [61]
**Immunoglobulin lambda chain**		X	-	Csősz et al. [61]
**Heat Shock protein-27 (HSP 27)**		X	-	Kim et al. [62]
**Beta-2 microglobulin (B2M)**		X	(Patent) [63]	Kim et al. [62] Maity et al. [64]
**Vascular endothelial growth factor (VEGF)**		X	-	Ang et al. [65]

* Glycomark^®^ assay kit is also useful to detect 1,5-AG in saliva. Halama et al. [56].

## 3. Sample Fluid

### 3.1. Urine

**Figure 1:**
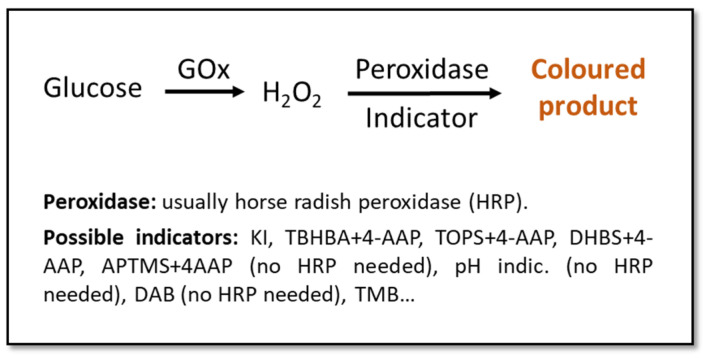
Schematic representation of classic enzymatic colorimetric methods for the detection of glucose. Indicators: potassium iodide (KI) [70,71,72,73,74,75,76,77], 2,4,6-tribromo-3-hydroxy benzoic acid (TBHBA) + 4-aminoantipyrine (4-AAP) [78,79], *N*-ethyl-N(3-sulfopropyl)-3-methyl-aniline sodium salt (TOPS) + 4-AAP [80], 3,5-dichloro-2-hydroxybenzenesulfonic acid (DHBS) + 4-AAP [81], 3-aminopropyltriethoxysilane (APTMS) + 4-AAP [82], pH indicator [83], 3,3′-diaminobenzidine (DAB) [84], 3,3′,5,5′-tetramethyl-benzidine (TMB) [81,85].

Urine is one of the best candidates as a diagnostic fluid for sensor arrays. Apart from permitting an easy, abundant, and non-invasive sampling, it is an aqueous solution (95% water) of inorganic salts containing, among others, urea. If obtained from a healthy individual, urine contains low concentration of lipids, proteins, and other high-molecular-weight compounds, which eases the detection of abnormally-high quantities of these big molecules [33,66]. The detection of glucose in urine is used for the diagnosis of DM, with the test being considered positive with glucose concentrations above ~100 mg/dL (5.6 mM) [67,68,69], especially if used together with parallel methods or as a part of a multi-sensing system. Most colorimetric glucose sensors rely upon enzyme-based reactions due to their high specificity and catalytic efficiency [70,71,72,73,74,75,76,77,78,79,80,81,82,83,84,85]. These enzymatic approaches usually involve glucose-oxidase (GOx), due to its specificity towards glucose and its tolerance for extreme pH, temperature, and ionic strength changes in comparison to other enzymes (Figure 1) [32,33,68,86]. Even if these tests might not be the most sensitive, they are a good alternative to invasive and more expensive/time-consuming methods for a fast and cost- effective screening [67,68].

Some research groups propose substituting enzymes with nanomaterials which can “mimic” enzymatic activity, and can help design more stable sensors and a more economic production of them [68]. For instance, in Yang et al. [67], the classic horseradish peroxidase (HRP) (Figure 1) is substituted by a bimetallic Fe-Pd nanoparticle (NP) coupled on the surface of reduced graphene. This achieved a limit of detection (LOD) of 1.76 µM, which is more than 3 orders of magnitude lower than the borderline concentration of glucose in the urine (Table 2). In Su et al. [69], HRP is again substituted, this time by Zn-Fe magnetic NPs. The latter approach shows an even better LOD of 0.3 µM. In both cases, the color change of the used indicator (3,3′,5,5′-tetramethylbenzidine, TMB) could be visible to the naked eye.

**Figure 2:**
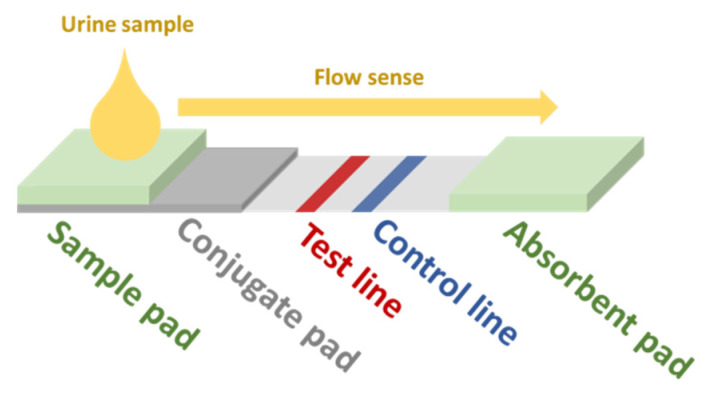
Scheme of a generic lateral-flow immunoassay paper-based sensor. Redrawn from Hainsworth et al. [19].

Another possible approach for the development of screening devices for relevant biomarkers in urine is the use of immunoassay-based sensors. In this kind of biosensor (generic scheme displayed in Figure 2), the sample is dropped in the sample pad. By capillarity, the fluid reaches the conjugate pad, which contains gold NPs (AuNPs) functionalized with the primary antibodies (Ab1) of the assay. The analyte in the sample binds to some of these Ab1. Some Ab1 remain free. All AuNPs follow the flow sense by capillarity until they get to the test line. Free Ab1 in the AuNPs bind to its antigen conjugates (Ag1) adhered in the test line, which brings up the characteristic red color of AuNPs. The rest of the AuNPs flow towards the control line, where the secondary Ab (Ab2) bind with the Ab1 bound to the analyte and the AuNPs, also resulting in a red color. The greater the amount of analyte, the lower the intensity of the red color at the test line. The absorbent pad eases the flow of the sample along the strip. This kind of sensor is very specific for the target analyte and could be implemented in a multi-sensing paper-based approach. In the recent work of Hainsworth et al., a lateral flow immunoassay paper sensor is presented for the potential screening of DR [19]. However, in this case, their target was 8-hydroxy-29-deoxyguanosine, which has been assessed to be a sensitive marker for DR [87,88]. For more insight into NP-based colorimetric immunoassay sensing, we refer the reader to the review of Ma et al. [89].

**Figure 3:**
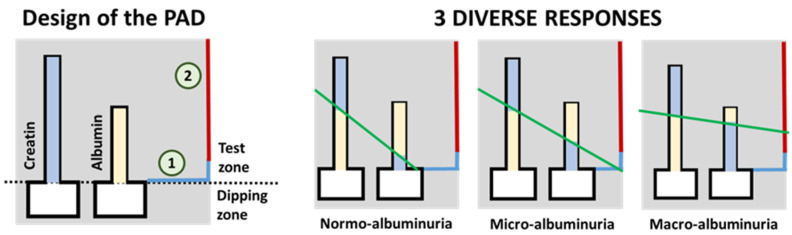
On the left, schematic representation of the paper-based sensor described in Hiraoka et al. [92] for the clinical assessment of albumin index (redrawn). On the right, three possible results for the test are shown. The green line represents a hand-drawn straight line that passes through the top of the two color-changed zones. Results (albuminuria index) are interpreted depending on which zone of the results chart (signaled as 1 or 2 in the scheme on the left) the drawn straight line crosses. For a better interpretation of the color references in this figure, the reader is referred to the web version of the article.

As stated by Sinha et al., the detection of albumin in the urine has been traditionally used as a screening method for diabetic nephropathy [90] and DR [23]. They forewarn about some drawbacks that may arise from using this method, such as late-detection or false negatives in some patients, but its great potential for its use in sensor-array screening devices should not be underestimated. For instance, Chaiyo et al. [91] describe a paper-based analytical device (PAD) for the colorimetric determination of the albumin (Alb) to creatinine (Cre) ratio (albuminuria index) in urine samples. The obtained LOD for Alb + Cre and Cre detection were 7.1 mg/dL and 5.4 mg/dL, respectively. Another particular approach has been recently presented by Hiraoka et al. [92], that introduce the concept of “drawing-PADs”. The PAD consists of two microchannels that change their color in the presence of the target analyte (either Alb or Cre), with the length of the color change being proportional to the concentration of analyte. These paper-based colorimetric sensors permit the semi-quantitative determination of the albuminuria index by hand-drawing a straight line crossing the color interfaces of the two microfluidic channels, as shown in Figure 3. The intercept of the resulting line (green line in the figure) with the color reference line (blue and red line on the right side of the schemes in the figure) provides the semi-quantitative detection of the albuminuria index (normo-albuminuria, micro-albuminuria, macro-albuminuria).

Measurement of ketones in the urine and blood is widely used for the diagnosis and monitoring of diabetic ketoacidosis (DKA). The acetoacetate (AcAc), acetone, and β-hydroxybutyrate (β-HB) ketonic bodies are catabolic products of free fatty acids. Ketone detection in urine has traditionally been performed via the qualitative or semi-quantitative analysis by means of dipsticks, impregnated with diverse reagents (e.g., nitroprusside reagents reacting with AcAc and acetone, but not with β-HB) [51]. During DKA, the ratio of β-HB:AcAc can be as high as 10:1, as β-HB is formed from the reduction of AcAc [93]. The assessment of this ratio is strongly relevant, as measuring only AcAc in urine would underestimate the extent of ketonemia [51]. Urine ketone determination is also limited by the paradoxical increase of urinary AcAc during resolution of ketoacidosis, which can be attributed to the conversion of excess β-HB back to AcAc [94]. Therefore, the measurement of urinary ketones, even if highly sensitive and non-invasive, is considered to be less accurate and specific than capillary β-HB [95]. Capillary β-HB testing is an accurate and precise method of early identification of DKA in the Emergency Department, which reduces unnecessary delays in the diagnosis of this condition. This test is more accurate than the assessment of urine ketones, and highly correlated with the clinical diagnosis of DKA [95]. Although the blood–retina barrier is less sensitive to presence and changes in DKA compared to the blood–brain barrier, it is still to be shown how DKA and its treatment affect DR development. 

Myoinositol (MI) is structurally similar to D-glucose, and is widely distributed in multiple organs. The reabsorption of MI in renal tubules competes with urinary glucose in cases of hyperglycemia, resulting in high concentrations of MI being excreted into the urine. It has been reported that urinary myoinositol (UMI) levels are increased in subjects with DM compared with controls. In healthy subjects, ~16–30 mg/day of MI is excreted in the urine, whereas, in subjects with DM, this level is increased to about 150~220 mg/day [96,97]. Takakado et al. [98] have conducted studies to establish a simple screening method for undiagnosed diabetes based on MI levels in urine samples collected at home.

Electronic noses (gas electrochemical sensor arrays) are also a promising technological platform for the diagnosis of T2D. Esfahani et al. [99] present electronic nose-based technologies that present a differentiated electrochemical response to urinary volatile organic compounds. After analyzing 140 urine samples from healthy and T2D patients and classifying the data using diverse ML approaches, they demonstrated that the developed tools discriminated between the two patient groups with an area under curve between 85–96%. A more recent publication [100] reaches 96–100% accuracy for T2D diagnosis exploiting the same electronic nose principle with urine samples, using both principal components analysis and an ML algorithm.

### 3.2. Saliva

Saliva is an aqueous solution (~99.5% water), containing mainly electrolytes, sugars, vitamins, proteins, and polypeptides [31,33,101]. It is a good candidate as a diagnostic fluid, since its sampling is easy (no need for trained personnel) and not invasive, can be collected in substantial volumes (0.1–7 mL/min) and is a less complex matrix in comparison to other body fluids (e.g., blood) [33,101]. However, the simplicity of saliva stems from its concentration, not from its composition; it has been shown to present more than 1000 diverse proteins in it, and biomarkers that have great potential for rapid test purposes [31,66,101]. There are pathological conditions that can modify the composition of the saliva [66]. In fact, several studies point at its potential for screening purposes and its successful use for testing several diseases (e.g., renal disease monitoring, human immunodeficiency virus, dental studies, or Cushing’s disease) [31,33,101,102]. Actually, a correlation between salivary and blood glucose levels in patients with and without DM has been shown [103], and saliva has already been declared as an excellent candidate for the monitoring of T2D [31,33].

Saliva glucose analysis has already been studied indeed, and some studies present interesting colorimetric approaches that might contribute to the design of multi-sensor DR screening approaches [33]. For instance, in Santana-Jiménez et al. [104], a colorimetric bi-enzymatic paper-based sensor for the detection of glucose in saliva is presented. It permits the detection of glucose by naked eye with a LOD of 0.84 mg/dL (46.6 µM) for real samples, which is approximately 2 orders of magnitude lower than the borderline concentration of glucose in saliva (~30 mg/dL, ~1.5 mM, Table 2) to be considered positive in a diabetes test. Moreover, they state good selectivity among other similar compounds (fructose and sucrose) and electrolytes (KCl and NaCl), also present in the saliva. Soni et al. [83] describe another paper-based colorimetric sensor, but instead of the classic GOx-HRP enzymatic method, a GOx-pH indicator approach is used. Its LOD is 22.2 mg/dL (1.2 mM), which is in the borderline for DM diagnosis (Table 2). This is not necessarily a drawback, as it could be applied in a qualitative binary-response sensor which, among parallel colorimetric data in the whole of a multi-sensing device, would be a valuable add on. Jahagirdar et al. [105] present a ML-based spectrophotometric device for the prediction of glucose level in blood from saliva samples that is low-cost, thus easing its widespread use.

**Figure 4:**
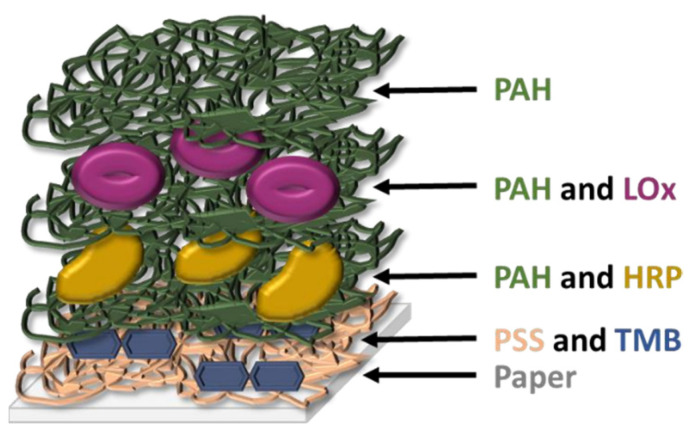
Schematic structure of the multi-layer sensor designed by Calabria et al. [113] (redrawn). PAH: poly(allylamine hydrochloride); PSS: poly(styrene sulfonate); LOx: L-lactate oxidase; HRP: horse radish peroxidase; TMB: 3,3′,5,5′-tetramethylbenzidine.

Takiyama et al. have shown that chronic hypoxia results from glycation of HbA1c and increased oxidative stress which captures oxygen, which is the cause of lactate increase in the blood [106]. The latter has also been recognized as a useful biomarker in saliva. This ion has already been used for the monitoring of DM due to the correlation between hyperlactatemia and this disease [107], and its concentration in saliva and blood are correlated (~1:4 ratio), as this ion passively diffuses from blood to the salivary glands [66,108]. Almost all lactate sensors tested with saliva samples found in literature are electrochemical [107,109,110] and, to our knowledge, Calabria et al. are the only ones presenting a colorimetric sensor for the detection of this ion in the saliva. They describe a reflectometric enzyme-based multi-layer PAD (Figure 4) containing all the reagents needed for the detection of lactate (lactate oxidase, HRP, and TMB as an indicator) with a LOD of 0.1 mM (900 mg/dL). They quantitatively determine the presence of lactate in the saliva by means of a smartphone, but a qualitative naked-eye interpretation could also be possible by using the adequate concentration of reagents. Other studies further describe the colorimetric sensing methods for lactose, but without using saliva as a working sample. For instance, Deng et al. [54] present another enzyme-based method, using GO@SiO_2_@CeO_2_ (GO: graphene oxide) nanosheets with intrinsic peroxidase activity, which permits avoiding the use of HRP. They successfully detected glucose, lactate, uric acid, and cholesterol simultaneously in serum and urine.

The degree of cortisol secretion is known to be related to the presence and number of DM complications [111]. Cortisol has also been one of the most widely studied salivary biomarkers of stress. Elevated cortisol production can lead to hypertension, central obesity, insulin resistance, and glucose intolerance [96]. Besides, studies in the past have demonstrated that elevated levels of inflammatory biomarkers, including C-reactive protein, tumor necrosis factor-α, interleukin-6, and interferon-γ in saliva can be found in obese/overweight children and adults [96]. In fact, salivary antioxidant status has been considered to be a measure of oxidative stress in individuals with T2D.

Malik et al. [112], driven by the strong potential of electrochemical variations in saliva for the detection of blood glucose, developed an electrochemical ML sensing technology that could use this physiological fluid for the assessment of glucose in blood. They reached an 85% accuracy, which is comparable to the accuracy of commercially available glucose-sensing dispositives. They also point out the possibility of miniaturizing these ion-selective sensor arrays for POC usage.

### 3.3. Breath

The analysis of breath for disease diagnosis is a practice used even in the time of Hypocrates, when it was found to be a useful method for monitoring human health [114]. Human breath is mainly composed of N_2_ (78%), O_2_ (16%), CO_2_ (4–5%), H_2_ (5%), inert gases (0.9%), and water vapor, but it also contains traces of inorganic (e.g., N_2_O, NO, or CO) and organic (e.g., acetone, ethanol, isoprene, or ethane) volatile species [114,115].

Acetone was first considered as a good breath-biomarker for DM in 1857 [116], and it is actually a good candidate, as its concentration in human breath appears to rise as the severity of DM increases, and there is a linear correlation between its concentration in blood and breath [114,117]. As shown in Table 1, patients without DM usually present breath acetone concentration lower than 2 ppm, while those having DM have values which can grow up to tenths of ppm [32]. Along with acetone, also isoprene (105 ppb in the breath of a person without DM) and aldehydes have proved to be good biomarkers for DM, and there are specific sensing approaches for acetone, aldehydes, and isoprene sensing, but they are not colorimetric or use gas samples other than breath as a proof of concept [114,118]. Nevertheless, Mazonne et al. [115,119] describe excitingly promising multi-sensing colorimetric PADs that change their color, showing different patterns depending on what gas mixture (i.e., breath) they are exposed to. In particular, they have been able to detect diverse types of lung cancer which were associated to a particular color pattern of the sensor arrays, acting like a ‘fingerprint’ of the disease. This ‘fingerprint’ multi-sensing concept is not new, and is closely related to the broadly-used and studied electronic tongues and noses [120,121,122], and opens up a world of low-cost user-friendly POC possibilities if applied to a naked-eye colorimetric approach.

Sarno et al. [123] propose an Arduino-assisted electronic nose capable of distinguishing among healthy (<120 mg/dL), pre-diabetic (120–150 mg/dL), and diabetic (>150 mg/dL) blood glucose concentrations by the analysis of the breath of the patient. The electrochemical data obtained from the sensor array was processed by a deep-learning classification method after optimization with discrete wavelet transform, and the output show an accuracy of 96.29%. A similar approach is also presented by Parte et al. [124], with a conductivity-based metal oxide gas sensor array and a modified deep learning convolution neural network algorithm integrated with support vector machines in order to detect acetone in breath and correlate it to DM. These are just two recent representative examples of the great potential of electronic noses (and gas sensor arrays in general) coupled to ML approaches for the non-invasive diagnosis of diabetes [125].

### 3.4. Tears

**Figure 5:**
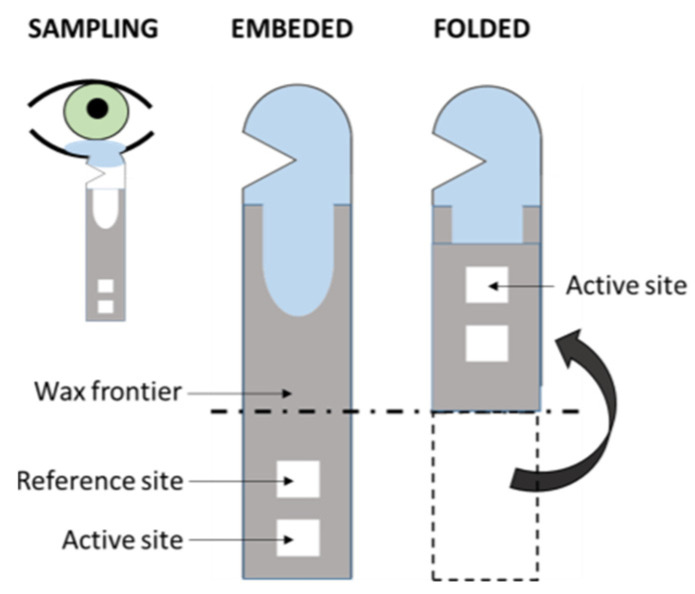
Scheme of the folding Schirmer strip sensor described in Kang et al. [130] (redrawn).

The volume of samples obtained from the tears by means of, for instance, a Schirmer strip (Figure 5), can be enough for a single-sensor method but, usually, slightly higher volumes would be needed in multi-array sensing in order to obtain high-enough sensitivity [66]. Moreover, the procurement of tears is a relatively non-invasive technique, as its sampling implies the use of cumbersome methods that might not be especially comfortable to the patient, yet easily approachable [83]. Beyond this, one should not avoid mentioning some selected works about this rich fluid that contains numerous analytes of great relevance for the assessment of the health status of the patient [32], including numerous proteins that permit proteomic approaches in the diagnosis of DR [47,61,126,127,128,129]. Kang et al. [130] reported a colorimetric Schirmer strip (which permits direct, rapid sampling) for the detection of glucose by the classic GOx-HRP bi-enzymatic method, previously mentioned (Figure 5). Gabriel et al. [81] and Moreira et al. [131] presented colorimetric, chitosan-modified paper-based sensors for the enzymatic detection of glucose. Both works are based upon the GOx-HRP method, and tear samples were previously obtained by means of borosilicate glass microcapillary tubes before dropping on the paper device. 

Prostaglandin E2 (also known as dinoprostone) is found in tears [132], and it is synthesized by the arachidonic acid and cyclooxygenase (COX) pathways. Up-regulation of COX2 occurs in retinal cells during the early onset of DR. Under these conditions, prostaglandin production is elevated, which in turn leads to an increased expression of vascular endothelial growth factor (VEGF), implicated in vascular leakage and neovascularization [133].

### 3.5. Sweat

Sweat might not be the best option for multi-array screening approaches; it seems that this body fluid could be more suitable for monitoring purposes, especially in the form of wearable electrochemical devices, which are usually interfaced with a ML software [134]. This approach can overcome the lack of big amounts of sample with direct contact of the sensor with the skin. In order to gain more insight into this complex fluid and its applications in diagnostics, we refer to the reviews from Senf et al. [66], Bandodkar et al. [135], and Kim et al. [136]. Additionally, an interesting microfluidic, colorimetric approach is presented by Choi et al. [137].

### 3.6. Other Samples

**Figure 6:**
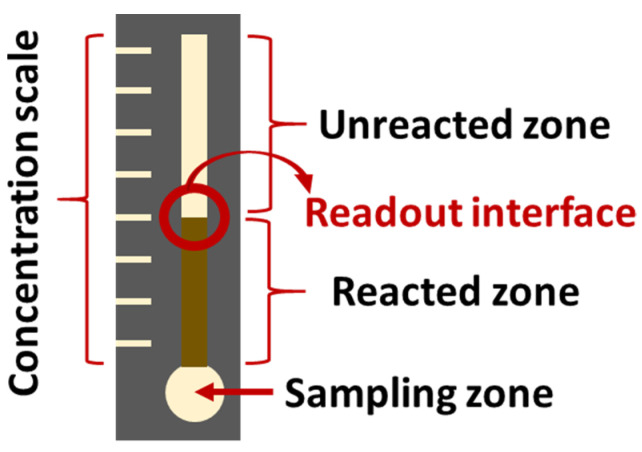
Schematic representation of the glucose sensor described by Wei et al. [84]. (redrawn).

There is a large amount of literature about the sensing of glucose, lactose, and other species that could act as biomarkers for DM. Although some of these studies do not test the sensors with urine, saliva, tear, sweat, or breath samples, their principles could potentially aid in the design of a POC multi-sensor device, and should not be overlooked [73,77,80,82,138,139,140,141,142,143,144]. Herein, a selection of articles is briefly described as a first approach to the extensive literature on the topic. Chiang et al. [145] present colorimetric PADs, whose hydrophobic frontier wax patterns were designed by means of a 3D printer. This procedure could reduce production time and cost. The described sensors showed a LOD of 0.3 mM for the detection of glucose and confirmed linearity between 0.5 and 4.5 mM, which would be sufficient for screening purposes with either urine, saliva, or tear samples. Li et al. [141] describe a colorimetric multi-sensing PAD which also uses a 3D printer for the delimitation of the wax-walled microfluidic channels. They simultaneously determine glucose, bovine serum albumin, and pH (proof of concept, no LOD reported) by means of a multichannel pattern. Another paper-based sensor for the detection of glucose (among others) is described in Wei et al. [84]. It consists in an enzymatic approach that relies in distance readout instead of color output, which makes it suitable for users with color blindness (Figure 6).

## 4. Sensor Array ML Technologies for DM and DR Screening

The diagnosis of a disease is a complex task involving a great number of factors, variability and uncertainty, and DM and DR are no exception. It is implausible to achieve a satisfactory verdict within an acceptable confidence range by relying upon a single factor. That is one of the multiple reasons why there is a growing interest in big-data studies (and their related computational methods). ML techniques, such as principal component analysis (PCA) and partial least squares (PLS), are already widely used to model and predict inherent correlations in complex biological data, as is the case of metabolomics and proteomics studies [128,146].

**Figure 7:**
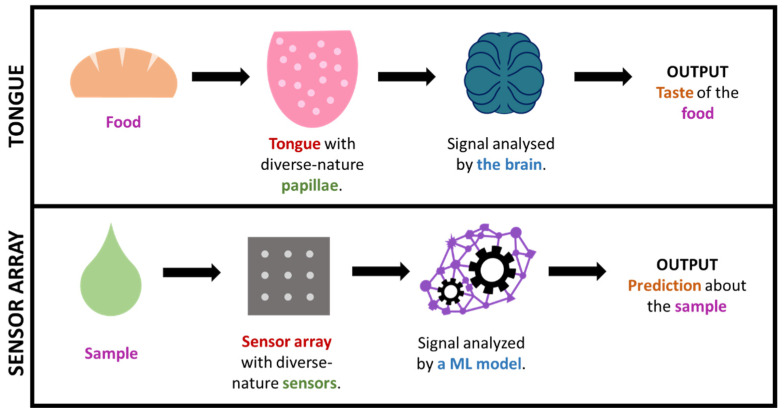
Simplified comparison of the sense of taste and a sensor array + ML technology.

In some cases, by using such big-data approaches, we are, in a way, mimicking what nature already does in order to interpret complex information. This is the case, for instance, for taste or smell, where a considerable amount of sensors presenting diverse responses to different odor/taste molecules produce a great amount of both relevant and irrelevant data, which creates a specific profile or ‘fingerprint’ that is latterly filtered and interpreted by the brain. This is how (generically speaking) some big-data/ML approaches work, including electronic noses/tongues [46,120,123,147,148,149,150]. It is worth highlighting that this fingerprint-based approach, which is commonly known as ‘non-targeted’, permits the gaining of useful output without the need of exactly knowing which specific analytes or processes occur in the sample (which tend to be markedly complex). Figure 7 illustrates the parallelism between taste and non-targeted sensing approaches.

ML computational strategies can be classified, for instance, in terms of supervision. Supervised models use labeled datasets that are employed for the training of the ML model. Once trained, the model is put into a test (validation phase) in order to assess its performance in predicting the label of ‘unknown’ (to the model, but not to the examiner’s) datasets. Depending on the selected datasets and labels, the ML model will be able to classify datasets into groups (e.g., distinguish datasets from patients with or without DM) or correlate dependent and independent variables, which permits the prediction of numerical values (e.g., sensing output and glucose concentration). Some of the most-used supervised models are PLS and its multiple variations, neural networks (e.g., artificial, convolutional, recurrent, or deep neural networks; ANN, CNN, RNN and DNN, respectively), support vector machine (SVM), or genetic algorithms (GA). On the other hand, unsupervised models analyze and cluster unlabeled datasets without the need for labeling (i.e., no human intervention needed during the analysis of the data). This is the case, for instance, the widespread PCA model, and the neural networks can also adopt to the unsupervised approach. In order to bypass patient-based variability and to reduce the number of false positives and negatives, we propose the reader to consider a supervised (classification-based) and non-targeted approach for the development of diagnostic technologies.

## 5. Special Mention to Paper-Based Supports

As the reader will have noticed, a great number of studies on paper-based sensors have been described in this review. The concept of PADs was first introduced by the Whitesides group in 2007 [151] and, since then, these devices have been widely used in several relevant fields, comprising clinical diagnostics [130] and, more specifically, in the detection of glucose [152]. The intrinsic microfluidic nature of cellulose nanofibers induces capillary force through the gaps of the material [130] which, together with the possibility of containing liquids in precise fluidic patterns [151], permits the design of multi-channel sensor arrays for the simultaneous sensing of diverse species at a time [153]. PADs can be fabricated in a fast and economic way, and can be easily designed to be accessible for non-trained personnel [154]. Moreover, they permit a wide range of detection approaches, including the user-friendly colorimetry [155,156]. Taking all of this into consideration, it is not surprising that a great percentage of POC sensing devices, especially those intended to be used in developing countries or low-resource environments, use this material as a support [35].

## 6. Conclusions

At present, it is unquestionable that ML modeling is one of the most promising and powerful tools for the development of diagnosis methods and technologies. It permits the fast cribbage and analysis of huge amounts of data from overwhelmingly complex biological matrices which, applied to diagnostics, can be translated into valuable support technologies that would ease rapid decision-making in early diagnosis and screening programs. We have seen that one can find a great number of colorimetric and electrochemical sensing methods for the detection of DM- and DR-related biomarkers, including some recent efforts towards the development of sensor-array technologies exploiting or not ML models for the sensing of diverse biomarkers and for diagnose purposes (including DM). With this review, we want to transmit our founded trust in ML-supported sensor arrays for the screening and early diagnosis of DM and DR, and we hope that the provided information and point of view will ease and encourage the reader to design and develop these promising technologies.

## Figures and Tables

**Table 2:** Acetone [32] and glucose concentrations (mg/dL [33] and mM [32] units) in diverse relevant physiological fluids from patients with or without diabetes. The pH of the fluid [32] and the time required to diffuse blood from the capillaries to the tissues (time lag) [33] are also shown.

Fluid	Non-Diabetic	Diabetic	pH	Time Lag
**Glucose**				
**Blood**	70–130 mg/dL 4.9–6.9 mM	36–720 mg/dL 2–40 mM	7.35–7.45	-
**Urine**	10.8–27.1 mg/dL 2.78–5.55 mM	50.1–100 mg/dL >5.55 mM	4.50–8.00	20 min
**Sweat**	1.1–1.98 mg/dL 0.06–0.11 mM	0.18–18.0 mg/dL 0.01–1 mM	4.60–6.80	20 min
**Saliva**	4.14–10.3 mg/dL 0.23–0.38 mM	9.91–31.9 mg/dL 0.55–1.77 mM	6.20–7.60	15 min
**Ocular fluids**	1.8–9.0 mg/dL 0.05–0.5 mM	9.01–90.1 mg/dL 0.5–5 mM	6.50–7.50	10 min
**Acetone**				
**Breath**	0.1–2 ppm	0.1–103.7 ppm	7.4–8.1	-

## Data Availability

Not applicable.
